# The health and well-being of adolescents: responding to their priorities

**DOI:** 10.1093/heapol/czag086

**Published:** 2026-09-30

**Authors:** David A Ross, Bhavya Nandini, Rajat Khosla, Anshu Mohan

**Affiliations:** Institute for Life Course Health Research, Stellenbosch University, 6th Floor, Clinical Building, Francie van Zijl Drive, Tygerberg 7505, South Africa; Partnership for Maternal, Newborn and Child Health (PMNCH), PMNCH Secretariat, World Health Organization, Avenue, Appia 20, CH 1211 Geneva 27,Switzerland; Partnership for Maternal, Newborn and Child Health (PMNCH), PMNCH Secretariat, World Health Organization, Avenue, Appia 20, CH 1211 Geneva 27,Switzerland; Family Health, Community and Life Course, World Health Organization (WHO), Regional Office for South-East Asia, World Health House, Indraprastha Estate, Mahatma Gandhi Road, New Delhi 110002, India

**Keywords:** adolescent health and well-being, multi-sectoral programming, youth priorities, return on investment, mental health financing, monitoring and accountability, low- and middle-income countries, commitments

## Abstract

Adolescents aged 10–19 years represent the largest cohort in human history and a critical global resource—yet realizing their potential requires deliberate, sustained investment in their health and well-being. This editorial introduces a six-article supplement in Health Policy and Planning addressing key priorities for adolescent health and well-being policy and programming, with particular attention to young people's own perspectives and evidence from low- and middle-income countries. The supplement opens with findings from the ‘What Young People Want Survey’, drawing on responses from over 1.5 million young people across 89 countries. Results reveal that adolescents’ priorities span multiple domains—especially learning and employability, safety and supportive environments, and health and nutrition—underscoring the necessity of multi-sectoral approaches. Three articles address financing. Together, they demonstrate that integrated health–education interventions can yield returns of at least 11 dollars per dollar invested and that scaling up treatment for adolescent depression and anxiety produces benefit–cost ratios of 15.4 and 13.9 in Colombia and South Africa, respectively—while cautioning that investments must be designed with an explicit equity focus to avoid deepening existing inequalities. Another article presents new World Health Organization and Partnership for Maternal Newborn and Child Health guidance for monitoring adolescent health and well-being holistically across the five domains of the UN conceptual framework, supported by an Excel-based data tool that maximizes use of existing data. The supplement concludes with an early assessment of commitments made at the 2023 Global Forum for Adolescents across four countries, finding that progress is hindered by fragmented systems, funding gaps, and insufficient disaggregated data. The evidence presented in this supplement makes clear that improving adolescent health and well-being is neither aspirational nor unaffordable—it is an investment with compelling returns. Realizing those returns demands that adolescents’ voices shape policy and that progress is rigorously monitored.

The current cohort of adolescents (10–19 years) is the largest the world has ever had (PMNCH et al. 2024). They represent a vital potential global talent resource for the coming decades. However, for this to be realized, today’s adolescents must enter adulthood healthy; well-educated; with a strong sense of belonging; motivated and empowered to make an active, positive contribution to society; and with the security and support that they need to reach their full potential (Ross et al. 2020). This will require governments and populations to consciously focus attention and resources on the health and well-being of adolescents (Partnership for Maternal Newborn & Child Health 2023a). Globally, this is increasingly recognized (Baird et al. 2025), and there is a growing consensus on the meaning of adolescent well-being (Ross et al. 2020) and the role everyone has to play to achieve adolescent health and well-being (WHO et al. 2023).

This supplement’s six articles highlight key issues that must be addressed if policies and programmes are to make a profound, positive impact on adolescent health and well-being over the coming decades. All the articles include young people as co-authors, and several draw on case studies conducted in low- or middle-income countries, ensuring that they are grounded in the everyday realities of young people.

Adolescents’ needs are always going to be greater than the resources available, and many stakeholders must have a say in what the priorities should be. However, the views of adolescents and young people themselves must be foremost. To this end, the article by Saran et al. (2026) reports the results of the ‘What Young People Want Survey’—a massive hybrid online/in-person survey that invited young people (10–24 years) to complete the statement: ‘To improve my well-being, I want…’. Responses were received from over 1.5 million young people from 89 countries. Almost all came from low- or middle-income countries. The responses were classified into five domains of adolescent well-being using natural language processing and word frequency analysis: good health and optimum nutrition; connectedness, positive values, and contribution to society; safety and a supportive environment; learning, competence, education, skills, and employability; and agency and resilience. The results showed that in all four World Health Organization (WHO) regions with more than 100 000 responses (Africa, South-East Asia, Eastern Mediterranean, and the Americas), young people’s responses encompassed all the five domains of well-being, especially learning, competence, education, skills, and employability (36.4%); safety and a supportive environment (28.4%); and good health and optimum nutrition (20.9%). These results should be reflected in the priorities that are set by country programmes for adolescents. The responses also emphasized the multi-faceted nature of adolescents’ ‘wants’ and that multi-sectoral programmes will be essential in order to meet them.

Three of the articles relate to the financing of programmes to promote adolescent well-being. The first, by Sheehan et al. (2026), states that major progress has been made in many areas of adolescent health and well-being. However, they emphasize that further progress is challenged by many factors, including the impact of climate change and of social media, rapid changes in skill requirements for employment and deep-seated inequalities within and between countries. There is now strong evidence of large economic and social returns that could be obtained from selected investments to promote adolescent health and well-being. However, the cost of these investments would be substantial, which will challenge many countries. Also, major inequalities within countries mean that countries need to plan carefully to ensure that investments do not exacerbate these inequalities. For example, if the quality of education is low for the poor but high for the rich, then further investment in education without an equity focus can perpetuate or deepen inequalities.

It is well-known that health and education are closely interconnected and that the relationship is bi-directional, with improvements in learners’ health enhancing their educational attainment and vice versa (Zimmerman et al. 2015). Using economic models, Symons et al. (2026) show that integrated health and education interventions can be an excellent economic investment, with at least 11 US dollars gained for every dollar invested.

The article by Sweeny et al. (2026) draws on national-level economic modelling studies in Colombia and South Africa to show that scaling up treatment for depression and anxiety among adolescents should yield excellent returns on the investment, with benefit–cost ratios for economic and social benefits estimated at 13.9 in South Africa and 15.4 in Colombia. However, they stress that to be able to implement these interventions at scale, both countries will need to train non-specialist providers, such as lay counsellors in schools and community settings. They also stress that national health information systems must be strengthened to provide age-disaggregated data on adolescent mental health and well-being.

Improvement in health and well-being is the ultimate goal of many adolescent policies and programmes. But how should such improvements be monitored? The article by Keogh et al. (2026) tackles this question and summarizes new guidance that has been produced by the WHO, the Partnership for Maternal, Newborn and Child Health, and partners to support countries in assessing adolescent health and well-being in a holistic, multi-sectoral way across the five domains of the United Nations conceptual framework on adolescent well-being. Rather than prescribing new indicators or data collection efforts, it provides a structured approach and an Excel-based data tool to improve the use of already existing data. It will also highlight data gaps.

The supplement concludes with an article by Mustafa et al. (2026). Twenty-four national governments, 3 regional organizations, 14 private sector or philanthropic organizations, 3 nongovernmental organizations, and 1 professional association made specific commitments related to adolescent well-being at the 2023 Global Forum for Adolescents (PMNCH 2023b). After mapping the nature of these commitments, this article provides an early assessment of actions taken by four countries (Bulgaria, Colombia, Malawi, and Nigeria) related to their commitments. The assessment used mixed methods, including a systematic review of national policies, strategic plans, legislative documents, progress reports, programme evaluations, and other official records, triangulated against key informant interviews to identify any discrepancies between stated policy intentions and implementation progress. This study showed that action has been taken related to the commitments in all four countries, but these efforts face persistent structural barriers, with challenges including fragmented health, education and social systems, funding gaps, and a lack of granular, usable data. It also identified that meaningful adolescent and youth engagement and multi-sectoral action were essential for impactful, equitable outcomes and that strong monitoring and evaluation, disaggregated data, and stable leadership are critical for sustained progress.

While much remains to be learned, this supplement provides important information on adolescent programming for policymakers and planners across the social sectors, including but not limited to the health sector. Critical insights include ‘What Young People Want’ to improve their well-being, guidance on how to measure and monitor adolescent health and well-being, and lessons from early assessments of the actions taken by four countries in Europe, South America, and Africa related to adolescent health and well-being commitments that they made in 2023. It also includes an important contribution to the growing evidence that carefully selected interventions to improve the health and well-being of adolescents can provide excellent social and economic returns on investment but that, to achieve meaningful results, such investments will need to be substantial.

Overall, the articles in the supplement spotlight four critical learnings. First, investment in the health and well-being of adolescents must be substantially increased. This is not only their right but will also bring substantial economic and social returns. Second, asking adolescents and young people themselves what they want to improve their health and well-being is essential, and their ‘wants’ should guide policies and programmes. Third, countries must monitor progress across all five domains of adolescents’ health and well-being. Luckily, although data gaps will be present, this is often going to be possible through better use of existing data. Fourth, countries must be held accountable for commitments that they have made through conducting periodic assessments of the actions that have been taken to fulfil those commitments. Throughout the world, the future of countries will depend on the health and well-being of today’s adolescents. The articles in this supplement make an important contribution to showing how this can be achieved.
